# Structure-Guided Design of C4-alkyl-1,4-dihydro-2*H*-pyrimido[4,5-*d*][1,3]oxazin-2-ones as Potent and Mutant-Selective Epidermal Growth Factor Receptor (EGFR) L858R/T790M Inhibitors

**DOI:** 10.1038/s41598-017-04184-9

**Published:** 2017-06-19

**Authors:** Yongjia Hao, Jiankun Lyu, Rong Qu, Deheng Sun, Zhenjiang Zhao, Zhuo Chen, Jian Ding, Hua Xie, Yufang Xu, Honglin Li

**Affiliations:** 1 State Key Laboratory of Bioreactor Engineering, Shanghai Key Laboratory of New Drug Design, School of Pharmacy, East China University of Science & Technology, Shanghai, 200237 China; 2 Division of Anti-tumor Pharmacology, State Key Laboratory of Drug Research, Shanghai Institute of Materia Medica, Chinese Academy of Sciences, Shanghai, 201203 China; 3 University of Chinese Academy of Sciences, Beijing, 100049 China

## Abstract

Epidermal growth factor receptor (EGFR) T790M acquired drug-resistance mutation has become a major clinical challenge for the therapy of non-small cell lung cancer. Here, we applied a structure-guided approach on the basis of the previous reported EGFR inhibitor (compound **9**), and designed a series of C4-alkyl-1,4-dihydro-2*H*-pyrimido[4,5-*d*][1,3]oxazin-2-one derivatives as novel mutant-selective EGFR inhibitors. Finally, the most representative compound **20a** was identified, which showed high selectivity at both enzymatic and cellular levels against EGFR^L858R/T790M^ (H1975 cell lines) over EGFR^WT^ (A431 cell lines). The representative compound **20a** also showed promising antitumor efficiency in the *in vivo* antitumor efficacy study of H1975 xenograft mouse model driven by EGFR^L858R/T790M^. These results provide a new scaffold for the treatment of dual-mutant-driven non-small cell lung cancer.

## Introduction

**Figure 1:**
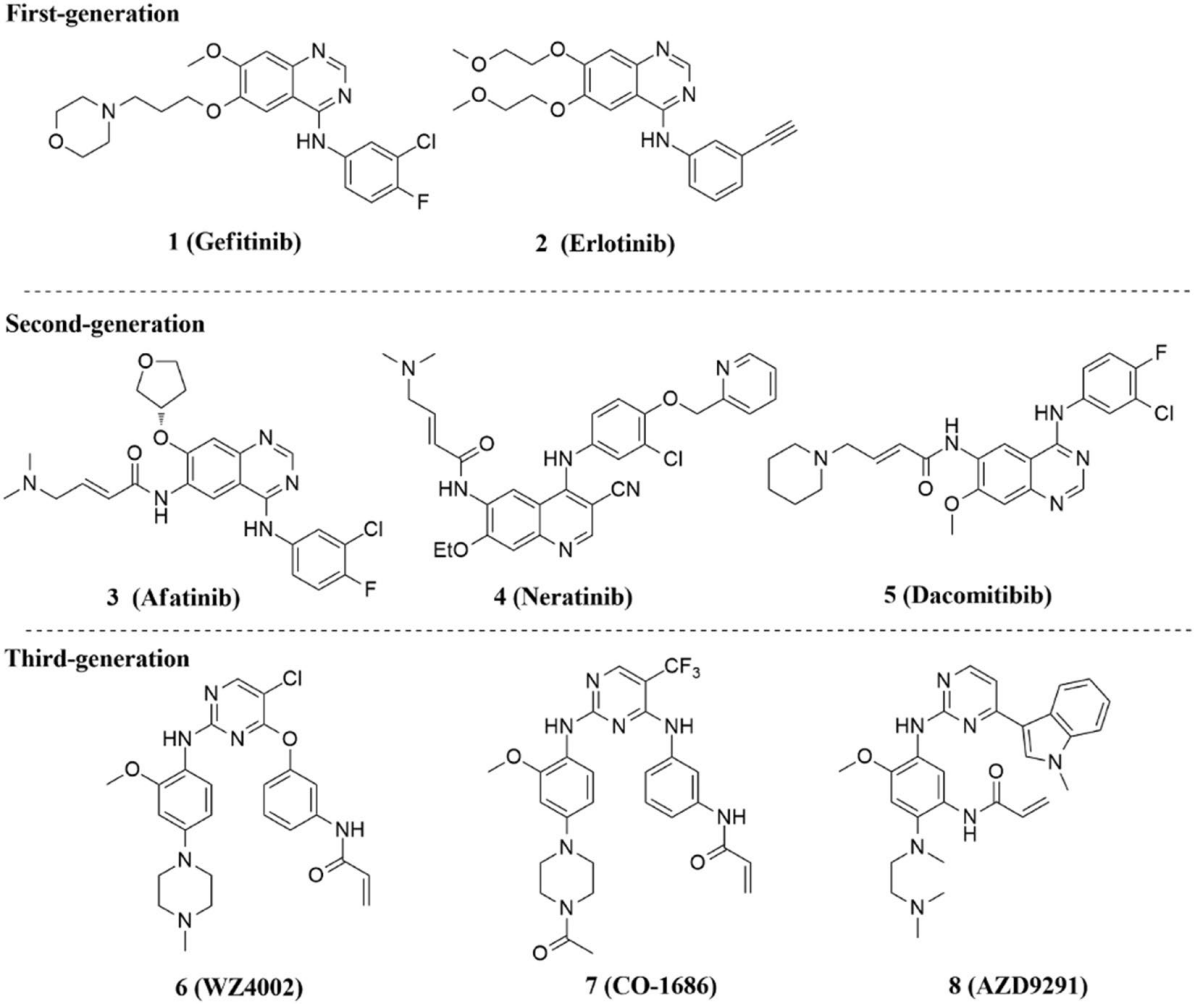
Structures of first-, second- and third-generation EGFR inhibitors.

The epidermal growth factor receptor (EGFR) is a member of ErbB (HER) receptor tyrosine kinases (RTKs) family, which plays essential roles in regulation of numerous normal physiological processes, such as cell proliferation, differentiation, metabolism and apoptosis^1, 2^. Conversely, overexpression and abnormal activation of EGFR is associated with varieties of human epithelial malignancies, especially non-small cell lung cancer (NSCLC). Thus, targeting EGFR has provided an effective anticancer strategy, and EGFR has become a well-established critical target for the treatment of NSCLC^3–5^. Whereafter, activating mutations within the EGFR catalytic domain have been successively discovered, of which, the exon 21 single point substitution mutation (L858R) and the exon 19 deletion (del E746-A750) are the two most prevalent activating mutations. Detection of EGFR activating mutations provides a useful marker for predicting the potential of first generation EGFR inhibitors^6–8^. Thus, compounds **1** (gefitinib) and **2** (erlotinib), two of the first-generation EGFR-targeted small molecule inhibitors (Fig. 1) have been used in clinic for the treatment of advanced NSCLC patients harboring these specific activating mutations. The two agents demonstrated remarkable therapeutic responses for these NSCLC patients, however, acquired drug-resistance often emerged after treatment of 10–14 months, which has become a major clinical challenge for the therapy of NSCLC^9–11^. The emergence of point mutations in the EGFR kinase domain is also closely related to acquired resistances, among which, the gatekeeper T790M secondary mutation (threonine790 → methionine790 mutation) is the primary mechanism of the acquired resistances, as it is the most common mutation and accounts for approximately 60% of all clinically observed acquired mutants^12^.

Extensive efforts have been devoted to the development of novel covalent EGFR inhibitors to overcome gefitinib- and erlotinib-resistant mutant (T790M mutation). These irreversible inhibitors are designed with electrophilic Michael-acceptor systems to covalently react with the conserved Cys797 in the EGFR active site, so as to increase inhibition potency against T790M mutant relative to reversible agents. Unfortunately, because of the dose-limiting toxicities attributed to inhibition of the wild-type (WT) EGFR, these second-generation irreversible inhibitors (Fig. 1) including **3** (afatinib)^13^, **4** (neratinib)^14^, **5** (dacomitinib)^15^ did not improve clinical efficacy for NSCLC patients who have developed T790M acquired resistance. Recently, the third-generation (mutant-selective) irreversible EGFR-tyrosine kinase inhibitors (TKIs) based on an amino pyrimidine scaffold, such as compounds **6** (WZ4002)^16^, **7** (CO-1686)^17^ and **8** (AZD9291)^18^ have demonstrated promising selectivity for EGFR^L858R/T790M^ mutant over WT EGFR, indicating that this strategy is feasible for overcoming EGFR T790M gatekeeper mutation in NSCLC treatment (Fig. 1). Based on their clinical significant benefits for NSCLC patients with EGFR T790M acquired drug-resistance mutation, United States Food and Drug Administration (FDA) has awarded compounds **7** and **8** “Breakthrough Therapy” designations in 2014^19^. Furthermore, **8** has been granted accelerated approval by FDA for the treatment of late-stage NSCLC patients with EGFR^T790M^ mutation-positive who have progressed after other EGFR TKIs therapy^20^.

**Figure 2:**
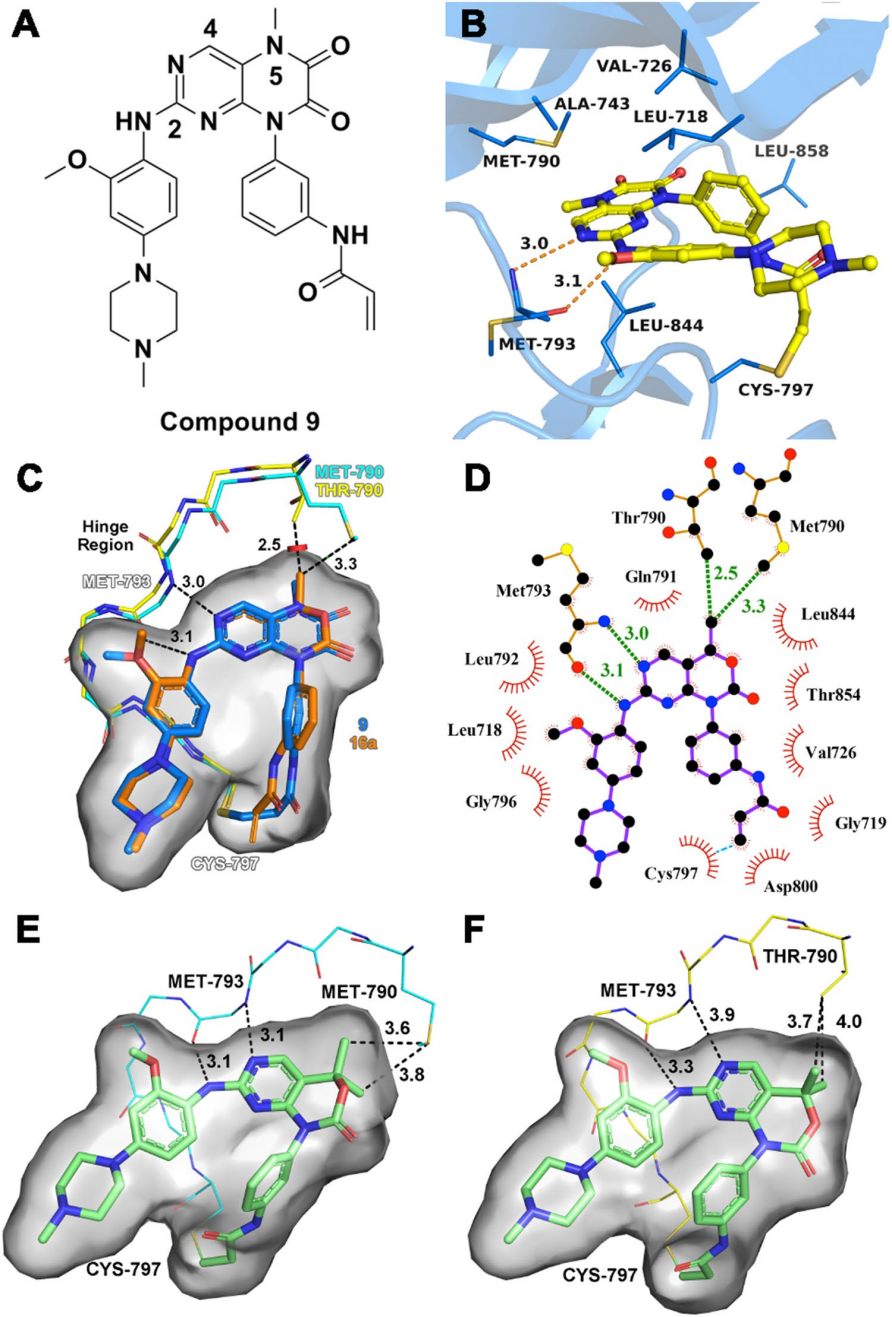
(**A**) The chemical structure of compound **9**. (**B**) The modeling binding mode of compound **9** bound to EGFR^T790M^ (PDB code: 3IKA). The sulfur atoms are colored in dark yellow and the carbon atoms are colored in light yellow. (**C**) The design strategy of C4-alkyl-1,4-dihydro-2*H*-pyrimido[4,5-*d*][1,3]oxazin-2-ones scaffold. Compound **16a** (orange) was aligned to the modeled conformation of compound **9** (marine) shown in panel A by SHAFTS. The EGFR^WT^ crystal structure (shown in yellow, PDB code: 4G5J) was aligned to the EGFR^T790M^ (shown in cyan, PDB code: 3IKA). The hinge regions of both EGFR^WT^ and EGFR^T790M^ were presented as stick. The carbon atoms of **9** are colored in marine and the nitrogen atoms of **9** are colored in dark blue. (**D**) The 2D diagram interaction of compound **16a**. (**E**) The proposed binding mode of compound **20a** bound to EGFR^T790M^ (cyan line, PDB code: 3IKA). (**F**) The proposed binding mode of compound **20a** bound to EGFR^WT^ (yellow line, PDB code: 4G5J). The conformations of compound **9**, **16a** and **20a** in the panel C,E and F are depicted as stick and highlighted in grey surface presentation.

In our previous studies to develop mutant-selective EGFR^L858R/T790M^ inhibitors, compound **9** was identified as a potent irreversible EGFR kinase inhibitor (Fig. 2A), which exhibited competitive enzymatic inhibitory activities against L858R/T790M mutant EGFR^21, 22^. In order to improve its cellular antiproliferative activity, meanwhile keep the selectivity profiles, we would like to describe the design and optimization of C4-alkyl-1,4-dihydro-2*H*-pyrimido[4,5-*d*][1,3]oxazin-2-one derivatives as novel mutant-selective EGFR inhibitors on the basis of compound **9**.

## Results

### Scaffold Hopping and Binding Mode Analysis

Using a structure-based approach, we reported compound **9** as a potent irreversible inhibitor with excellent selectivity against EGFR^L858R/T790M^ kinase over EGFR^WT^ kinase (4.7 nM vs 474 nM, 101-fold)^21^. As shown in Fig. 2B, the modeling binding mode suggests that compound **9** binds to the EGFR^T790M^ (PDB code: 3IKA) via covalently modifying the conserved Cys797 residue at the lip of the ATP-binding site. The 6,7-dioxo-6,7-dihydropteridine core is predicted to interact with the residue Met793 at the hinge region by an expected bidentate hydrogen bonding and also form a hydrophobic sandwich with the ceiling residue Ala743 and the floor residue Leu844. The N-methylpiperazine ring faces to the solvent exposed region. The two phenyl rings substituted at C2 and N8 adapt a U-shape binding mode. These interactions suggested by the docking study stabilize the basic recognition of inhibitor-kinase complex. Moreover, the methyl group at N5-position points to the mutant gatekeeper Met790 residue and strengthens nonpolar contacts with T790M mutants, which is a vital contribution proposed by the docking study to the selectivity against EGFR^L858R/T790M^ over EGFR^WT^. Besides, the L858R mutation is far from the ATP-binding pocket of EGFRs and would contribute little to the interaction at the inhibitor-binding site. Given the former successful design strategy targeting EGFR^L858R/T790M^ and aimed at improving selectivity over EGFR^WT^, a scaffold hopping of the 6,7-dioxo-6,7-dihydropteridine core was conducted. The piperazinediones part of compound **9** was replaced with 1,3-oxazinan-2-one in order to introduce one or two hydrophobic substituents at the C4-position, and thus it could not only occupy the lipophilic subpocket formed by the gatekeeper residue but also strengthen nonpolar contacts with Met790 residue (Fig. 2C and D). This computer-aided approach resulted in compound **16a**. As expected, the superposition of compound **9** and compound **16a** demonstrates that the C4-position methyl group is in van der Waals contacts (shown in little green disc, distance of 3.3 Å) with the side chain of Met790 residue in the case of the EGFR^T790M^ while for the EGFR^WT^ the corresponding distance is much closer (shown in large red disc, distance of 2.5 Å), resulting in a steric clash between the N5-methyl group and side chain of Thr790 residue. These different interactions between N5-position moiety and the gatekeeper residue would affect the potency and selectivity.

### Chemistry

**Figure 3:**
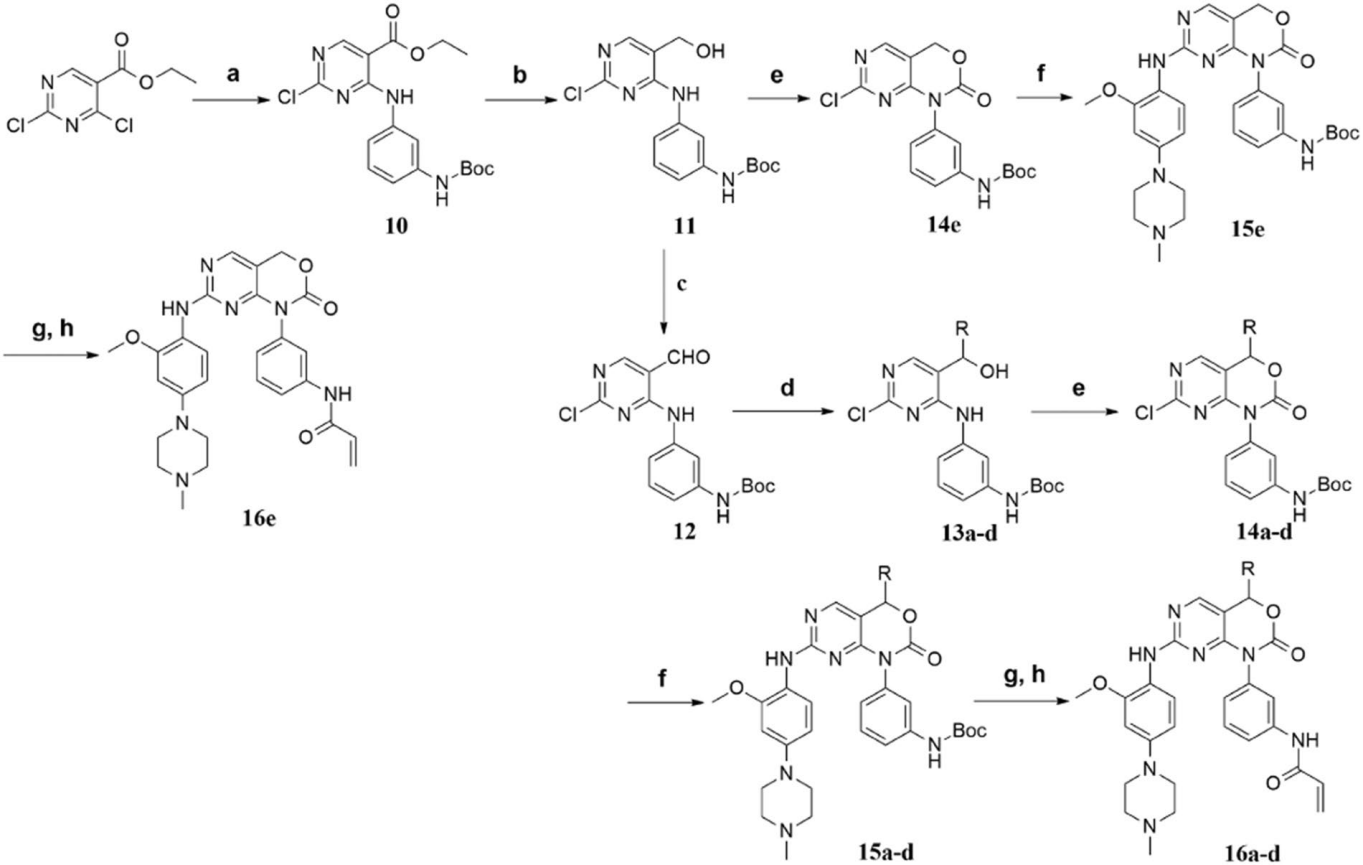
Synthesis of the Target Compounds **16a**-**e** ^*a*^. ^*a*^Reagents and conditions: (**a**) *tert*-butyl(3-aminophenyl)carbamate, *N*,*N*-diisopropylethylamine (DIPEA), acetonitrile (CH_3_CN), reflux, 6 h, 86%; (**b**) LiAlH_4_, dry tetrahydrofuran (THF), 0 °C, 4 h, 15%; (**c**) MnO_2_, CH_2_Cl_2_, r.t., overnight, 82%; (**d**) RBrMg, dry THF, 0 °C, 5–8 h, 74–82%; (**e**) 1,1′-carbonyldiimidazole, K_2_CO_3_, dry CH_3_CN, r.t., overnight, 69–75%; (**f**) arylamine, trifluoroacetic acid, isopropanol, reflux, overnight, 31–39%; (**g**) trifluoroacetic acid, CH_2_Cl_2_, r.t., 5 h, 77–83%; (**h**) acrylyl chloride, DIPEA, CH_2_Cl_2_, 0 °C to r.t., overnight, 35–49%.

The synthesis of compounds **16a**-**e** is outlined in Fig. 3. In summary, treatment of the ethyl 2,4-dichloropyrimidine-5-carboxylate with Boc-protected amine yielded the ethyl4-((3-((*tert*-butoxycarbonyl)amino)phenyl)amino)-2-chloropyrimidine-5-carboxylate (**10**), followed by reduction of the ethyl ester group and oxidization of the hydroxymethyl to obtain the key intermediate (**12**). A reaction of **12** with Grignard reagents generated compounds **13a**-**d**. Then compounds **11**, **13a**-**d** were cyclized with 1,1′-carbonyldiimidazole to obtain the ring-closure compounds (**14a**-**e**), which were reacted with arylamine by displacement of 2-chloro in the pyrimidine to yield compounds **15a**-**e**. Subsequently, removal of the protecting group with trifluoroacetic acid in dichloromethane and reacted with acryloyl chloride to provide the desired compounds **16a**-**e**.

**Figure 4:**
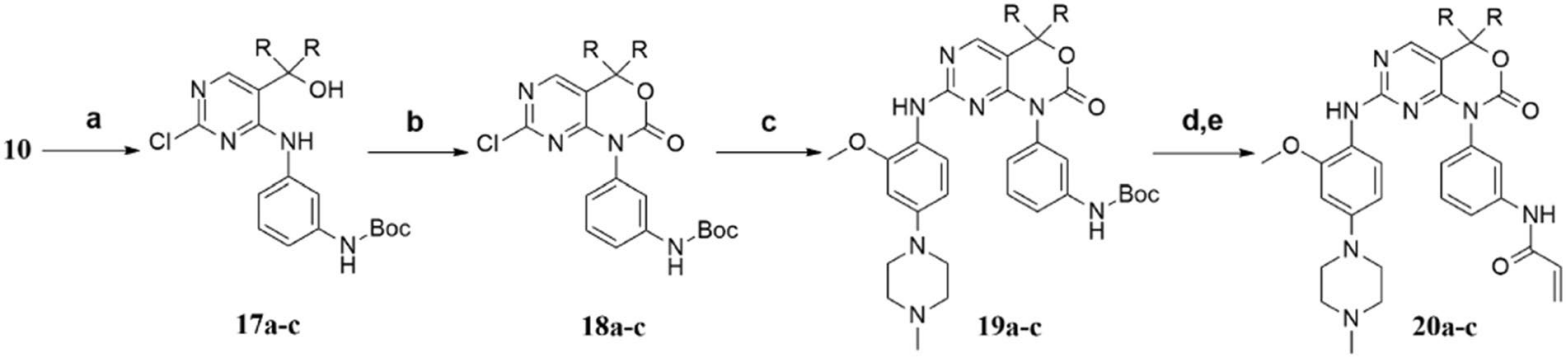
Synthesis of the target compounds **20a**-**c** ^*a*^. ^*a*^Reagents and conditions: (**a**) RBrMg, dry THF, 0 °C, 8–10 h, 33–48%; (**b**) 1,1′-carbonyldiimidazole, K_2_CO_3_, dry CH_3_CN, r.t., overnight, 65–85%; (**c**) arylamine, trifluoroacetic acid, isopropanol, reflux, overnight, 52–61%; (**d**) trifluoroacetic acid, CH_2_Cl_2_, r.t., 5 h, 78–83%; (**e**) acrylyl chloride, DIPEA, CH_2_Cl_2_, 0 °C to r.t., overnight, 45–51%.

Synthesis of compounds **20a**-**c** is shown in Fig. 4. Treatment of compound **10** with appropriate Grignard reagents afforded the corresponding intermediates **17a**-**c**, which were performed by a procedure analogous to that for synthesis of compound **16a** to give the desired compounds **20a**-**c**.

### *In Vitro* Structure-activity Relationship (SAR) and Structural Modification

**Figure 5:**
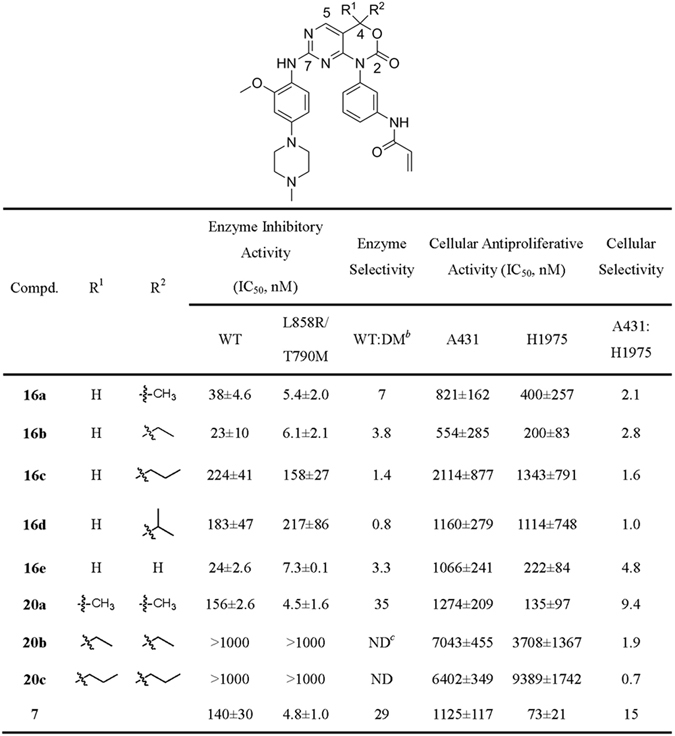
*In Vitro* Enzyme Inhibitory Activities and Cellular Antiproliferative Activities of Compounds 16a-e, 20a-c^*a*^. ^*a*^Kinase activities were determined through using the ELISA-based EGFR-TK assay. *In vitro* antiproliferation activities of the compounds were examined through using the sulforhodamine B (SRB) colourimetric assay. IC_50_ values are averages of at least three independent determinations and reported as the means ± standard deviations (SD). ^*b*^Dual-mutant (EGFR^L858R/T790M^). ^*c*^Not determined.

Initially, a series of 1,4-dihydro-2*H*-pyrimido[4,5-*d*][1,3]oxazin-2-one derivatives (**16a**-**d**) that contain a single hydrophobic group (methyl, ethyl, propyl and isopropyl) at the C4-position of the scaffold were designed and synthesized (Fig. 3). Their *in vitro* enzymatic inhibitory activities against EGFR^L858R/T790M^ and EGFR^WT^ were evaluated by using the well-established ELISA-based assay. As shown in Fig. 5, compounds **16a** and **16b** indeed demonstrated different inhibitory activities for dual-mutant (DM) and WT EGFR kinases. They displayed single nanomolar inhibitory activities for EGFR^L858R/T790M^ with IC_50_ values of 5.4 and 6.1 nM, respectively, while their inhibition for EGFR^WT^ were ~4–7-fold less potent. Introduction of propyl and isopropyl groups in the 4-position of the core led to compounds **16c** and **16d**, which showed decreased potency for EGFR kinases and significant loss in selectivity profiles between EGFR^L858R/T790M^ and EGFR^WT^ (Fig. 5). The bioactivities of **16c** and **16d** indicated that this hydrophobic subpocket is unable to accommodate these two longer and bulkier alkyl groups, thus resulting in detrimental influence on potency and selectivity. To validate the key contribution of the introduced alkyl groups for EGFR kinases selectivity, compound **16e**, a C4-unsubstituted analogue, was also prepared (Fig. 3). Compared to compound **16a**, compound **16e** displayed not only less potent inhibition effect for EGFR^L858R/T790M^ (IC_50_ = 7.3 nM), but also slight improvement in inhibitory activity for EGFR^WT^ (IC_50_ = 24 nM), leading to a 3.3-fold decrease in its selectivity between the DM and WT EGFRs. These results therefore showed that the small hydrophobic C4-substitutent, such as methyl group, occupying the lipophilic subpocket formed by the mutant gatekeeper residue was favorable to inhibitor of DM EGFR with selectivity over WT EGFR.

On the basis of above single-substituent structural modification, we hypothesized that introducing one more suitable hydrophobic moiety at C4-position to further strengthen the nonpolar contacts with mutant gatekeeper Met790 may be beneficial for improving the mutant selectivity profile. So we applied this rationale to compound **16a** through attaching a dual-alkyl group at the C4-position, and resulted in compounds **20a**-**c** (Fig. 4). As expected, compound **20a**, dual-methyl groups substituted derivative at C4-position of the 1,4-dihydro-2*H*-pyrimido[4,5-*d*][1,3]oxazin-2-one, inhibited EGFR^L858R/T790M^ with an IC_50_ value of 4.5 nM, which was similar to that of compound **16a**. Whereas its inhibition of WT EGFR was less potent (IC_50_ = 156 nM). Thus, it showed approximate 35-fold selectivity between EGFR^L858R/T790M^ and EGFR^WT^, and was highly comparable to the positive control compound **7**. The structure-based insights from molecular modeling are consistent with the experimental observations. As shown in the Fig. 2E, compound **20a** was covalently docked into the ATP-binding site of EGFR^T790M^ and the hinge binder part is predicted to tightly interacts with the backbone of Met793 (two H-bonds distance: 3.1 and 3.1 Å). Its upper methyl group fits the methionine gatekeeper pocket well and its lower methyl group is involved in van der Waals contacts with Met790. In the case of the proposed binding mode of EGFR^WT^ (Fig. 2F), the branched residue Thr790 leads a steric clash to the dual methyl moiety (Fig. 2C and D) and pushed the core of compound **20a** to the right side of the ATP-binding site, which attenuates the hydrogen-bonding with the hinge region (two H-bonds distance: 3.3 and 3.9 Å). These comparable docking studies also validated this selectivity profile. However, when introducing bulkier substitutions, such as dual-ethyl and dual-propyl groups at the C4-position of the core, the compounds (**20b** and **20c**) led to a complete loss of inhibitory activities (>1000 nM) for both DM and WT EGFR kinases. These results implied that longer and bulkier substituents than dual-methyl groups are not tolerated to the hydrophobic subpocket.

### *In Vitro* Cellular Anti-proliferation Assay

Having explored potent inhibition and obvious selectivity of EGFR kinases by introduction of alkyl groups to C4-position to the 1,4-dihydro-2*H*-pyrimido[4,5-*d*][1,3]oxazin-2-one ring, we subsequently evaluated their anti-proliferative activities in H1975 cells (expressing EGFR^L858R/T790M^) and A431 cells (expressing EGFR^WT^) *in vitro* by using the sulforhodamine B (SRB) colourimetric assay. The anti-proliferative activities of our designed compounds against H1975 and A431 cell lines range from 135 nM to more than 9 μM and from 554 nM to more than 7 μM, respectively (Fig. 5). Additionally, the selectivity at the cellular level is from 0.7 to 9.4-fold. Importantly, compound **20a** exhibited potent growth-suppressing activities against the H1975 cell lines with IC_50_ values of 135 nM, and achieved high selectivity over A431 cell lines (9.4-fold, IC_50_ = 1274 nM), which is comparable with the positive control compound **7** (15-fold). Therefore, compound **20a**, which shows best potency at both enzymatic and cellular levels, was selected as the representative compound evaluated in the following pharmacology assays.

### Western Blot Analysis

**Figure 6:**
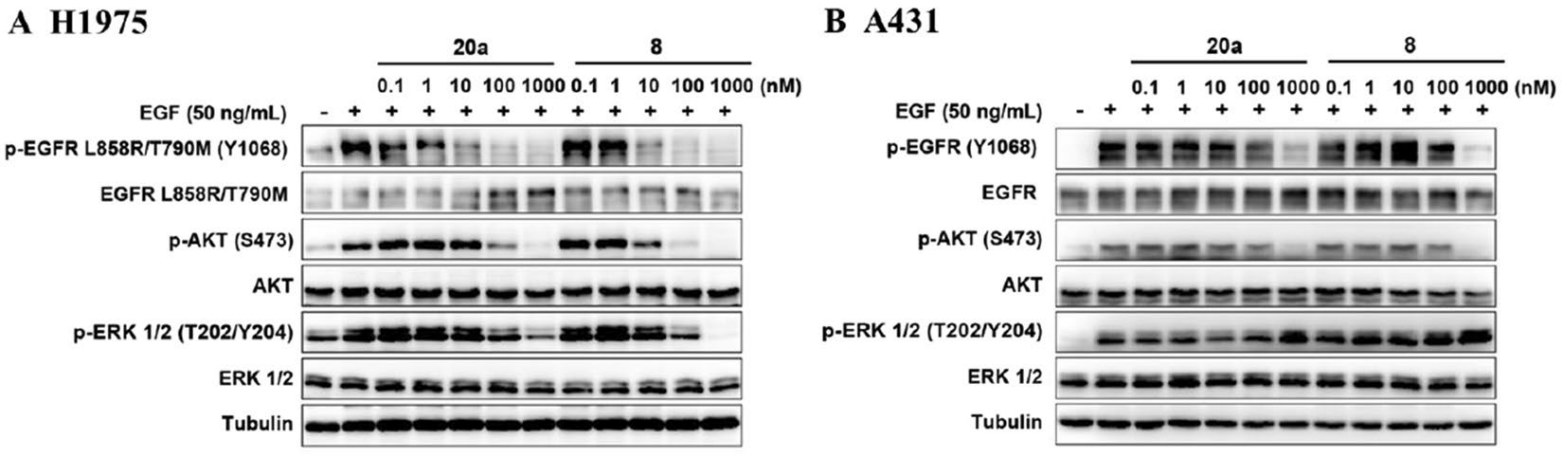
Compound **20a** inhibits phosphorylation of EGFR and downstream signaling substrates in (**A**) H1975 cell line harboring EGFR^L858R/T790M^, while less potent in (**B**) A431 cells harboring EGFR^WT^. Cells were starved in serum-free medium for 24 h before incubation of indicated concentrations of compounds **20a** and **8** (positive control) and harvested after stimulation by EGF (50 ng/mL) for 10 min. Phosphorylation of EGFR and downstream signaling substrates was examined by Western Blot analysis. Blot source data is provided in Supplementary Fig. S1.

In order to further validate the observed selectivity of enzymatic and cellular inhibition, we selected the representative compound **20a** to examine its suppressive functions on the activation of EGFR and the downstream signaling proteins in H1975 and A431 cell lines. As illustrated in Fig. 6A, compound **20a** inhibited the phosphorylation of EGFR^L858R/T790M^ and AKT in H1975 cells at the concentration as low as 10 nM, and inhibited downstream molecule ERK phosphorylation at the concentration up to 100 nM, demonstrating similar activity with compound **8** (positive control). Meanwhile, its inhibitory activity against A431 cell lines harboring EGFR^WT^ was obviously less potent (Fig. 6B). For example, compound **20a** significantly suppressed the activation of EGFR^L858R/T790M^ in H1975 cells at 10 nM, but it did not show obvious effect until reaching high concentration at 1 μM in A431 cells. The results demonstrated that **20a** strongly inhibited EGFR^L858R/T790M^ while EGFR^WT^ sparing at the cellular level, further suggesting that **20a** was a potent and selective EGFR^L858R/T790M^ inhibitor.

### *In Vivo* Antitumor Efficacy Study of Compound 20a

**Figure 7:**
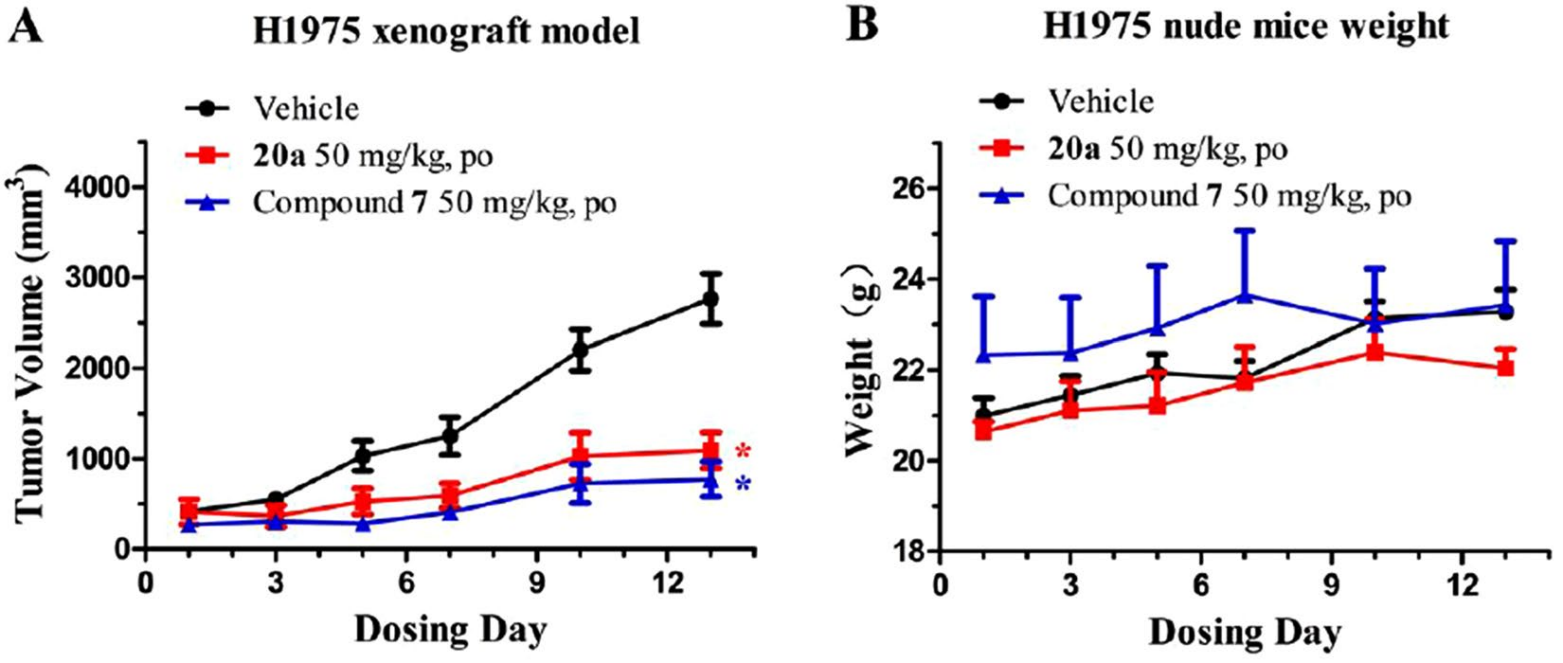
Preliminary *in vivo* antitumor efficacy of compound **20a** against H1975 NSCLC xenograft mouse model. H1975 cells were implanted as described in Methods. Compounds **20a** and **7** were administered orally at a concentration of 50 mg/kg/day for consecutive 14 days starting from day 0. H1975 xenograft mouse model tumor volumes (**A**) and body weights (**B**) were recorded every two or three days. All values represent mean ± SEM. The tumor growth inhibition (TGI, %) was measured on the final day of the treatment (drug-treated group versus the vehicle control), where the asterisk ∗ represents P < 0.05 (Student’s t test) compared to the vehicle group.

In view of its encouraging ability of inhibition for EGFR kinases and cells, we also evaluated the preliminary *in vivo* antitumor activity of compound **20a** in H1975 xenograft mouse model driven by EGFR^L858R/T790M^ (Fig. 7). Compound **20a** and positive control compound **7** were administered orally once per day at a dosage of 50 mg/kg for continuous two weeks (14 days). As shown in Fig. 7A, compound **20a** (tumor growth inhibition (TGI) = 71%, P < 0.05) potently suppressed the proliferation and growth of the tumor, which is comparable with that of compound **7** (TGI = 72%, P < 0.05). Furthermore, the dosage of compound **20a** was well tolerated, with no obvious effect on the body weight observed during the treatment period (Fig. 7B).

## Discussion

Herein, we combined the ligand-based scaffold hopping with the structure-based analysis of differences of the binding pockets between EGFR^L858R/T790M^ and EGFR^WT^ to design a new 1,4-dihydro-2*H*-pyrimido[4,5-*d*][1,3]oxazin-2-one scaffold targeting EGFR^L858R/T790M^ and improve selectivity over EGFR^WT^. The scaffold hopping method *in silico* helps us modify more than one hydrophobic substituent at C4-position. Compared with the compound **16e** (no substitution at C4), the single methyl group substituent on the compound **16a** occupies hydrophobic subpocket, leading to a slightly 1.4-fold increase of the enzymatic inhibition against EGFR^L858R/T790M^ (7.3 nM vs 5.4 nM). With the slight potency decline against EGFR^WT^ (**16e** vs **16a**, 24 nM vs 38 nM), the selectivity against EGFR^L858R/T790M^ over EGFR^WT^ boost 2.1-fold (**16e** vs **16a**, 3.3-fold vs 7-fold). In the second round dual-substituent optimization, the one more methyl group at C4-position stabilized the nonpolar interaction with gatekeeper residue Met790 (Fig. 2E), keeping the enzymatic inhibitory activities against EGFR^L858R/T790M^ at the same level (**16a** vs **20a**, 5.4 nM vs 4.5 nM). Besides, dual-methyl group introduces more steric clashes into the interaction between the inhibitor and the EGFR^WT^ (Fig. 2F), leading to a pronouncing decrease of enzymatic inhibition against EGFR^WT^ (**16a** vs **20a**, 38 nM vs 156 nM). Thus, the representative compound **20a** achieved the best selectivity (35-fold) in this series, which is comparable with the compound **7** (29-fold). The selectivity improvements achieved along the whole structural optimization verified the rationality of our design strategy.

**Figure 8:**
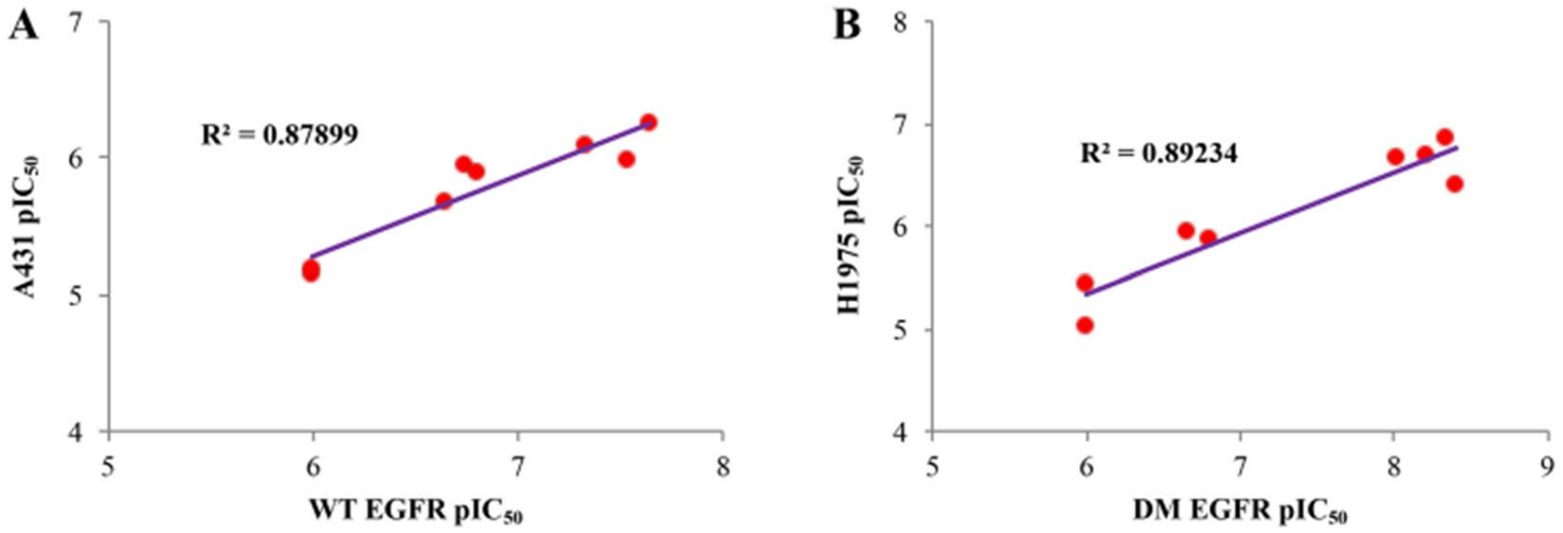
(**A**) Linear relationship between pIC50s against EGFR^WT^ and pIC50s against A431 cell lines. (**B**) Linear relationship between pIC50s against EGFR^DM^ and pIC50s against H1975 cell lines.

According to the results from enzymatic and cellular inhibition assays and western blot analysis, this series of compounds present good targeting properties. In particular, the antiproliferation potencies against H1975 and A431 cell lines are highly correlated with the enzymatic inhibitory activities against EGFR^L858R/T790M^ and EGFR^WT^, respectively (Fig. 8). The western blot analysis of representative compound **20a** validated the selectivity profile observed both in the enzymatic and cellular assays (Fig. 6). Moreover, the *in vivo* efficacy for double-mutant tumor xenografts of **20a** is highly comparable with that of the drug at clinical trials (compound **7**). These good targeting and selective properties of representative compound may imply the potential clinical use for the treatment of the drug-resistance NSCLC and less side-effects caused by the EGFR inhibitor without mutant selectivity.

In summary, through structure-guided design approach based on our previous work on mutant-selective EGFR inhibitor (compound **9**), we have designed and generated a novel 1,4-dihydro-2*H*-pyrimido[4,5-*d*][1,3]oxazin-2-one scaffold, followed by introduction of alkyl groups contacting the Met790 gatekeeper residue into the C4-position to keep the selectivity for the EGFR^L858R/T790M^ over EGFR^WT^, leading to discovery of one of the most potent compound **20a**. **20a** not only showed remarkable selectivity of enzyme inhibition for EGFR^L858R/T790M^ over EGFR^WT^, but also strongly inhibited the proliferation of H1975 cells, while significantly less effective on A431 cell lines. A further preliminary *in vivo* antitumor efficacy evaluation on **20a** suggested that it significantly inhibited tumor growth in H1975 NSCLC xenograft mouse model via po dosing at 50 mg/kg/day for 14 days. Considering the encouraging enzymatic and cellular selectivity and *in vivo* antitumor efficiency of compound **20a**, this agent might be a promising lead compound for further optimization as a selective EGFR^L858R/T790M^ inhibitor to overcome T790M resistance mutation. The results also provide more insights for designing new classes of mutant-selective EGFR inhibitors. Further pre-clinical evaluations for the candidate compound are in progress and will be performed in due course.

## Materials and Methods

### Molecular Modeling

The X-ray structures (PDB code: 3IKA and 4G5J) were downloaded from the Protein Data Bank (PDB, http://www.pdb.org). The comparative docking study was conducted via three steps: First, compounds **9** and **20a** were docked into the EGFR^T790M^ (PDB code: 3IKA) and EGFR^WT^ (PDB code: 4G5J) as reversible inhibitors using Glide^23^ (version 5.5) in extra precision (XP) mode with default settings; Then, the covalent bond between the Cys797 and the electrophilic group was formed by the build panel in Maestro^24^; Finally, to fix the bond lengths and eliminate steric clashed, the covalently modified inhibitor-kinase complexes were minimized in water solvent using MacroModel application with flexible ligand and constrained protein.

The scaffold hopping was conducted by SHAFTS^25, 26^. The query molecule is the docked pose of compound **9** obtained from the above docking study. The aligned pose of compound **16a** (shown in Fig. 2C) was generated by fast 3D similarity calculation of SHAFTS. The score for the similarity calculation between compound **9** and **16a** is 1.494. The range of the SHATFS’ similarity score is from 0 (totally dissimilar) to 2 (completely similar). The Fig. 2D was generated by LigPlot+^27^.

### Reagents and General Methods

All chemical reagents and solvents were purchased from commercial sources, and used without further purification. The positive control compound **7** was purchased from Shanghai Future Chemical Technology Co., Ltd. Thin-layer chromatography (TLC) was used to monitor the progress of reactions. Column chromatography was performed using 300−400 mesh silica gel (Qingdao Haiyang Chemical Co.,Ltd). ^1^H and ^13^C NMR spectra were recorded on a Bruker AV-400 spectrometer at 400 MHz and 100 MHz, respectively. LC-MS and high-resolution mass spectra (HRMS) were measured at the Institute of Fine Chemistry of East China University of Science and Technology. The purity of all final compounds were analyzed by using HPLC (Hewlett-Packard 1100), with the purity of all compounds to be over than 95% (>95%). HPLC instrument: (column: Zorbax RP-18, 5 μm, 4.6 mm × 250 mm, reverse phase; detector: photodiode array detector). The mobile phases A and B were acetonitrile and 10 mM NH_4_OAc in water (pH 6.0), respectively. A gradient of 5−100% the mobile phase A was run at a flow rate of 1.0 mL/min.

### Ethyl-4-((3-((*tert*-butoxycarbonyl)amino)phenyl)amino)-2-chloropyrimidine-5-carboxy late (10)

A mixture of ethyl-2,4-dichloropyrimidine-5-carboxylate (22.100 g, 100 mmol), 3-(*tert*-Butoxycarbonylamino)aniline (20.800 g, 100 mmol) and DIPEA (12.900 g, 100 mmol) in CH_3_CN (220 mL) was heated with reflux for 6 h. After cooling to room temperature, the white solid was collected by filtration and washed with CH_3_CN to give the product **10** (33.719 g, 86%). ^1^H NMR (400 MHz, DMSO-*d*_6_) δ 10.23 (s, 1H), 9.50 (s, 1H), 8.80 (s, 1H), 7.70 (s, 1H), 7.35 (d, *J* = 7.6 Hz, 1H), 7.29 (t, *J* = 8.0 Hz, 1H), 7.24 (d, *J* = 8.4 Hz, 1H), 4.39 (q, *J* = 7.2 Hz, 2H), 1.49 (s, 9H), 1.36 (t, *J* = 7.2 Hz, 3H). LC-MS: m/z: 393.1 (M+H)^+^.

### *tert*-Butyl(3-((2-Chloro-5-(hydroxymethyl)pyrimidin-4-yl)amino)phenyl)carbamate (11)

To a solution of compound **10** (31.360 g, 80 mmol) in dry THF (150 mL) was dropwise added LiAlH_4_ (2.5 M in THF, 80 mL) at 0 °C. The reaction mixture was stirred for 4 h at 0 °C and was then treated with saturated aqueous NH_4_Cl. The solid was filtered off, and the filtrate was extracted with ethyl acetate (3 × 100 mL). The organic layer was washed with saturated aqueous NaCl, dried over anhydrous Na_2_SO_4_ and concentrated in vacuo. The residue was purified by silica gel chromatography (petroleum ether/ethyl acetate = 2:1, v/v) to give the product **11** (4.209 g, 15%). ^1^H NMR (400 MHz, CDCl_3_) δ 8.38 (s, 1H), 7.87 (s, 1H), 7.70 (s, 1H), 7.34 (d, *J* = 8.4 Hz, 1H), 7.25 (t, *J* = 8.0 Hz, 1H), 7.05 (d, *J* = 8.0 Hz, 1H), 6.66 (s, 1H), 4.65 (s, 2H), 1.52 (s, 9H). LC-MS: m/z: 351.1 (M+H)^+^.

### *tert*-Butyl(3-((2-Chloro-5-formylpyrimidin-4-yl)amino)phenyl)carbamate (12)

To a solution of compound **11** (3.500 g, 10 mmol) in dichloromethane (30 mL) was added activated manganese (IV) oxide (58%, 15.000 g, 100 mmol). The mixture was stirred overnight at room temperature. The solid was filtered off through Celite, and the filtrate was concentrated under reduced pressure. The resulting crude product was purified by silica gel chromatography (petroleum ether/ethyl acetate = 4:1, v/v) to give the product **12** (2.850 g, 82%). ^1^H NMR (400 MHz, CDCl_3_) δ 10.60 (s, 1H), 9.89 (s, 1H), 8.56 (s, 1H), 7.82 (t, *J* = 2.0 Hz, 1H), 7.39 (d, *J* = 8.8 Hz, 1H), 7.31 (t, *J* = 8.0 Hz, 1H), 7.20 (d, *J* = 8.0 Hz, 1H), 6.58 (s, 1H), 1.53 (s, 9H). LC-MS: m/z: 349.1 (M+H)^+^.

### *tert*-Butyl(3-((2-Chloro-5-(1-hydroxyethyl)pyrimidin-4-yl)amino)phenyl)carbamate (13a)

To a solution of **12** (1.044 g, 3 mmol) in dry THF (15 mL) was dropwise added a solution of methylmagnesium bromide (1 M in THF, 9 mL) under the condition of argon gas at 0 °C. The mixture was stirred for 5 h at 0 °C, and was then quenched with a saturated aqueous NH_4_Cl solution and extracted with ethyl acetate (80 mL). The organic layer was washed with saturated aqueous NaCl, dried over anhydrous Na_2_SO_4_ and evaporated to obtain a residue. Purification by silica gel chromatography (petroleum ether/ethyl acetate = 2:1, v/v) to give the product **13a** (0.896 g, 82%). ^1^H NMR (400 MHz, CDCl_3_) δ 8.83 (s, 1H), 7.77 (s, 1H), 7.70 (s, 1H), 7.29 (d, *J* = 8.4 Hz, 1H), 7.05 (d, *J* = 8.0 Hz, 1H), 6.67 (s, 1H), 4.87 (q, *J* = 6.4 Hz, 1H), 1.55 (d, *J* = 6.4 Hz, 3H), 1.52 (s, 9H). LC-MS: m/z: 365.1 (M+H)^+^.

### *tert*-Butyl(3-(7-Chloro-4-methyl-2-oxo-2*H*-pyrimido[4,5-*d*][1,3]oxazin-1(4*H*)-yl)phenyl) carbamate (14a)

To a mixture of **13a** (0.815 g, 2.2 mmol), potassium carbonate (0.455 g, 3.3 mmol) in dry CH_3_CN (10 mL) was added 1,1′-carbonyldiimidazole (1.069 g, 6.6 mmol). The reaction mixture was stirred overnight at room temperature. The reaction mixture was poured into water, and extracted with ethyl acetate. The organic layer was washed with saturated aqueous NaCl, dried over anhydrous Na_2_SO_4_ and concentrated in vacuo. The residue was purified by silica gel chromatography (petroleum ether/ethylacetate = 2:1, v/v) to give the product **14a** as a white solid (0.642 g, 75%). ^1^H NMR (400 MHz, DMSO-*d*_6_) δ 9.59 (s, 1H), 8.54 (s, 1H), 7.61 (s, 1H), 7.41–7.36 (m, 2H), 6.98 (d, *J* = 6.8 Hz, 1H), 5.86 (q, *J* = 6.4 Hz, 1H), 1.74 (d, *J* = 6.4 Hz, 3H), 1.47 (s, 9H). LC-MS: m/z: 391.1 (M+H)^+^.

### *tert*-Butyl(3-(7-((2-Methoxy-4-(4-methylpiperazin-1-yl)phenyl)amino)-4-methyl-2-oxo-2*H*-pyrimido[4,5-*d*][1,3]oxazin-1(4*H*)-yl)phenyl)carbamate (15a)

To a mixture of **14a** (0.737 g, 1.89 mmol), 2-methoxy-4-(4-methylpiperazin-1-yl)aniline (0.502 g, 2.27 mmol) in isopropanol (15 mL) was added trifluoroacetic acid (210 μL, 2.84 mmol). The reaction solution was heated with reflux for 10 h. After cooling to room temperature, the mixture was neutralized with saturated aqueous NaHCO_3_, and extracted with CH_2_Cl_2_. The organic layer was washed with saturated aqueous NaCl, dried over anhydrous Na_2_SO_4_ and concentrated in vacuo. The residue was purified by silica gel chromatography (ethylacetate/methanol = 25:1, v/v) to give the product **15a** (0.403 g, 37%). ^1^H NMR (400 MHz, CDCl_3_) δ 8.46 (s, 1H), 8.18 (d, *J* = 8.8 Hz, 1H), 7.79 (s, 1H), 7.74 (s, 1H), 7.24–7.20 (m, 3H), 6.69 (s, 1H), 6.54 (d, *J* = 2.4 Hz, 1H), 6.50 (dd, *J* = 8.8 Hz, *J* = 2.4 Hz, 1H), 4.52 (q, *J* = 6.4 Hz, 1H), 3.85 (s, 3H), 3.17 (t, *J* = 4.8 Hz, 4H), 2.60 (t, *J* = 4.8 Hz, 4H), 2.36 (s, 3H), 1.51 (s, 9H), 1.49 (d, *J* = 6.4 Hz, 3H). LC-MS: m/z: 576.3 (M+H)^+^.

### *N*-(3-(7-((2-Methoxy-4-(4-methylpiperazin-1-yl)phenyl)amino)-4-methyl-2-oxo-2*H*-pyrimido[4,5-*d*][1,3]oxazin-1(4*H*)-yl)phenyl)acrylamide (16a)

To a solution of **15a** (0.277 g, 0.48 mmol) in dichloromethane (5 mL) was added trifluoroacetic acid (1 mL). The mixture was stirred for 5 h at room temperature. The reaction solution was neutralized with saturated aqueous NaHCO_3_, and extracted with CH_2_Cl_2_. The organic layer was washed with saturated aqueous NaCl, dried over anhydrous Na_2_SO_4_ and concentrated in vacuo to obtain a white solid (0.187 g, 82%), which was used in the next reaction without further purification.

To a solution of the above obtained compound (0.187 g, 0.39 mmol), DIPEA (0.076 g, 0.59 mmol) in CH_2_Cl_2_ (5 mL) was added acryloyl chloride (42 μL, 0.51 mmol) dissolved in CH_2_Cl_2_ (1 mL) at 0 °C. The reaction mixture was stirred overnight at room temperature. The mixture was partitioned between dichloromethane (50 mL) and saturated aqueous NaHCO_3_ (5 mL). The organic layer was washed with saturated aqueous NaCl, dried over anhydrous Na_2_SO_4_ and concentrated in vacuo. The residue was purified by silica gel chromatography (dichloromethane/methanol = 20:1, v/v) to give the product **16a** (0.075 g, 36%). ^1^H NMR (400 MHz, DMSO-*d*_6_) δ 10.36 (s, 1H), 8.22 (s, 1H), 7.84 (d, *J* = 8.0 Hz, 1H), 7.77 (s, 1H), 7.67 (s, 1H), 7.47 (t, *J* = 8.0 Hz, 1H), 7.25 (d, *J* = 8.4 Hz, 1H), 7.10 (d, *J* = 7.6 Hz, 1H), 6.52–6.50 (m, 1H), 6.44 (dd, *J* = 16.8 Hz, *J* = 10.0 Hz, 1H), 6.26 (dd, *J* = 16.8 Hz, *J* = 1.6 Hz, 1H), 6.08–6.06 (m, 1H), 5.77 (dd, *J* = 10.4 Hz, *J* = 1.6 Hz, 1H), 5.72 (q, *J* = 6.4 Hz, 1H), 3.75 (s, 3H), 3.03–3.01 (m, 4H), 2.44–2.42 (m, 4H), 2.22 (s, 3H), 1.69 (d, *J* = 6.4 Hz, 3H). ^13^C NMR (100 MHz, DMSO-*d*_6_) δ 163.21, 159.03, 156.44, 153.15, 150.51, 139.69, 136.47, 131.68, 129.28, 127.11, 124.36, 120.18, 118.96, 106.32, 106.01, 99.73, 72.39, 55.57, 54.58, 48.61, 45.70, 19.65. HRMS(ESI) (m/z): (M+H)^+^ calcd for C_28_H_32_N_7_O_4_ 530.2516, found, 530.2512. HPLC purity: 98.48%, retention time = 8.71 min.

The following compounds **16b**-**e** were prepared by a method similar to that for the synthesis of compound **16a**.

### *N*-(3-(4-Ethyl-7-((2-methoxy-4-(4-methylpiperazin-1-yl)phenyl)amino)-2-oxo-2*H*-pyrimido[4,5-*d*][1,3]oxazin-1(4*H*)-yl)phenyl)acrylamide (16b)

^1^H NMR (400 MHz, DMSO-*d*_6_) δ 10.46 (s, 1H), 8.22 (s, 1H), 7.85 (d, *J* = 8.4 Hz, 1H), 7.81 (s, 1H), 7.66 (s, 1H), 7.47 (t, *J* = 8.0 Hz, 1H), 7.26 (d, *J* = 8.4 Hz, 1H), 7.09 (d, *J* = 8.0 Hz, 1H), 6.55 (d, *J* = 2.0 Hz, 1H), 6.48 (dd, *J* = 16.8 Hz, *J* = 6.8 Hz, 1H), 6.27 (dd, *J* = 16.8 Hz, *J* = 1.6 Hz, 1H), 6.10–6.09 (m, 1H), 5.77 (dd, *J* = 10.0 Hz, *J* = 1.6 Hz, 1H), 5.55 (t, *J* = 6.8 Hz, 1H), 3.76 (s, 3H), 3.19 (t, *J* = 4.4 Hz, 4H), 2.92 (t, *J* = 4.4 Hz, 4H), 2.56 (s, 3H), 2.09–1.95 (m, 2H), 1.03 (t, *J* = 7.2 Hz, 3H). ^13^C NMR (100 MHz, DMSO-*d*_6_) δ 163.29, 156.45, 153.58, 150.39, 139.79, 136.41, 131.76, 129.26, 127.06, 124.28, 120.74, 120.08, 119.02, 106.62, 104.38, 100.11, 76.93, 55.65, 53.18, 47.17, 27.36, 8.50. HRMS(ESI) (m/z): (M+H)^+^ calcd for C_29_H_34_N_7_O_4_ 544.2672, found, 544.2654. HPLC purity: 99.34%, retention time = 9.37 min.

### *N*-(3-(7-((2-Methoxy-4-(4-methylpiperazin-1-yl)phenyl)amino)-2-oxo-4-propyl-2*H*-pyrimido[4,5-*d*][1,3]oxazin-1(4*H*)-yl)phenyl)acrylamide (16c)

^1^H NMR (400 MHz, DMSO-*d*_6_) δ 10.45 (s, 1H), 8.22 (s, 1H), 7.85 (d, *J* = 8.0 Hz, 1H), 7.80 (s, 1H), 7.66 (s, 1H), 7.47 (t, *J* = 8.0 Hz, 1H), 7.25 (d, *J* = 8.4 Hz, 1H), 7.08 (d, *J* = 8.0 Hz, 1H), 6.54 (d, *J* = 2.0 Hz, 1H), 6.48 (dd, *J* = 16.8 Hz, *J* = 10.0 Hz, 1H), 6.26 (dd, *J* = 16.8 Hz, *J* = 1.6 Hz, 1H), 6.10–6.09 (m, 1H), 5.77 (dd, *J* = 9.6 Hz, *J* = 1.6 Hz, 1H), 5.60 (t, *J* = 7.2 Hz, 1H), 3.76 (s, 3H), 3.14 (t, *J* = 4.4 Hz, 4H), 2.79 (t, *J* = 4.4 Hz, 4H), 2.47(s, 3H), 2.00–1.92 (m, 2H), 1.56–1.44 (m, 2H), 0.99 (t, *J* = 7.2 Hz, 3H). ^13^C NMR (100 MHz, DMSO-*d*_6_) δ 163.27, 156.39, 153.45, 150.36, 139.78, 136.42, 131.75, 129.26, 127.04, 124.29, 120.60, 120.10, 119.01, 106.55, 104.77, 100.01, 75.68, 55.63, 53.53, 47.54, 36.18, 17.23, 13.55. HRMS(ESI) (m/z): (M+H)^+^ calcd for C_30_H_36_N_7_O_4_ 558.2829, found, 558.2836. HPLC purity: 97.05%, retention time = 11.98 min.

### *N*-(3-(7-((2-Methoxy-4-(4-methylpiperazin-1-yl)phenyl)amino)-2-oxo-4-isopropyl-2*H*-pyrimido[4,5-*d*][1,3]oxazin-1(4*H*)-yl)phenyl)acrylamide (16d)

^1^H NMR (400 MHz, DMSO-*d*_6_) δ 10.52 (s, 1H), 8.22 (s, 1H), 7.87 (d, *J* = 8.0 Hz, 1H), 7.85 (s, 1H), 7.64 (s, 1H), 7.47 (t, *J* = 8.0 Hz, 1H), 7.26 (d, *J* = 7.6 Hz, 1H), 7.05 (d, *J* = 7.6 Hz, 1H), 6.57 (d, *J* = 1.6 Hz, 1H), 6.50 (dd, *J* = 16.8 Hz, *J* = 10.0 Hz, 1H), 6.26 (dd, *J* = 16.8 Hz, *J* = 1.6 Hz, 1H), 6.14–6.10 (m, 1H), 5.77 (dd, *J* = 10.0 Hz, *J* = 1.6 Hz, 1H), 5.40 (d, *J* = 4.8 Hz, 1H), 3.77 (s, 3H), 3.18 (t, *J* = 4.4 Hz, 4H), 2.74 (s, 3H), 2.27–2.22 (m, 1H), 1.04 (d, *J* = 6.8 Hz, 3H), 0.99 (d, *J* = 6.8 Hz, 3H). ^13^C NMR (100 MHz, DMSO-*d*_6_) δ 163.33, 156.37, 154.32, 150.33, 139.87, 136.31, 131.77, 129.29, 127.08, 124.14, 120.98, 119.88, 119.03, 106.77, 103.07, 100.29, 80.57, 55.70, 52.39, 46.36, 33.25, 17.87, 15.96. HRMS(ESI) (m/z): (M+H)^+^ calcd for C_30_H_36_N_7_O_4_ 558.2829, found, 558.2831. HPLC purity: 99.04%, retention time = 11.62 min.

### *N*-(3-(7-((2-Methoxy-4-(4-methylpiperazin-1-yl)phenyl)amino)-2-oxo-2*H*-pyrimido[4,5-*d*][1,3]oxazin-1(4*H*)-yl)phenyl)acrylamide (16e)

^1^H NMR (400 MHz, CDCl_3_+CD_3_OD) δ 8.09 (s, 1H), 7.89 (s, 1H), 7.75 (d, *J* = 8.4 Hz, 1H), 7.47 (t, *J* = 8.0 Hz, 1H), 7.42–7.39 (m, 1H), 7.07 (d, *J* = 7.6 Hz, 1H), 6.42–6.38 (m, 2H), 6.15 (d, *J* = 7.2 Hz, 1H), 5.71 (dd, *J* = 9.2 Hz, *J* = 2.8 Hz, 1H), 5.36 (s, 2H), 3.80 (s, 3H), 3.37 (t, *J* = 4.8 Hz, 4H), 3.24 (t, *J* = 4.8 Hz, 4H), 2.82 (s, 3H). ^13^C NMR (100 MHz, CDCl_3_+CD_3_OD) δ 161.37, 156.64, 155.73, 149.09, 143.64, 140.07, 135.12, 135.07, 133.55, 131.59, 128.51, 126.93, 124.71, 124.63, 124.02, 112.78, 105.19, 105.14, 69.18, 59.60, 57.68, 51.93, 47.34. HRMS(ESI) (m/z): (M+H)^+^ calcd for C_27_H_30_N_7_O_4_ 516.2359, found, 516.2364. HPLC purity: 95.07%, retention time = 10.60 min.

### *tert*-Butyl(3-((2-Chloro-5-(2-hydroxypropan-2-yl)pyrimidin-4-yl)amino)phenyl)carbama te (17a)

To a solution of compound **10** (3.920 g, 10 mmol) in dry THF (25 mL) was dropwise added a solution of methylmagnesium bromide (1 M in THF, 30 mL) under the condition of argon gas at 0 °C. The mixture was stirred for 8 h at 0 °C, and was then quenched with a saturated aqueous NH_4_Cl solution and extracted with ethyl acetate (100 mL). The organic layer was washed with saturated aqueous NaCl, dried over anhydrous Na_2_SO_4_ and evaporated to obtain a residue. Purification by silica gel chromatography (petroleum ether/ethyl acetate = 2.5:1, v/v) to give the product **17a** (1.811 g, 48%). ^1^H NMR (400 MHz, DMSO-*d*_6_) δ 10.01 (s, 1H), 9.41 (s, 1H), 8.12 (s, 1H), 7.62 (t, *J* = 2.0 Hz, 1H), 7.40 (dd, *J* = 8.0 Hz, *J* = 1.2 Hz, 1H), 7.25 (t, *J* = 8.4 Hz, 1H), 7.13 (d, *J* = 8.8 Hz, 1H), 6.43 (s, 1H), 1.56 (s, 6H), 1.48 (s, 9H). LC-MS: m/z: 379.1 (M+H)^+^.

### *tert*-Butyl(3-(7-Chloro-4,4-dimethyl-2-oxo-2*H*-pyrimido[4,5-*d*][1,3]oxazin-1(4*H*)-yl)phen yl)carbamate (18a)

To a mixture of **17a** (1.512 g, 4 mmol), potassium carbonate (0.828 g, 6 mmol) in dry CH_3_CN (20 mL) was added 1,1′-carbonyldiimidazole (1.296 g, 8 mmol). The reaction mixture was stirred overnight at room temperature. The reaction mixture was poured into water, and extracted with ethyl acetate (3 × 25 mL). The organic layer was washed with saturated aqueous NaCl, dried over anhydrous Na_2_SO_4_ and concentrated in vacuo. The residue was purified by silica gel chromatography (petroleum ether/ethylacetate = 2.5:1, v/v) to give the product **18a** (1.049 g, 65%). ^1^H NMR (400 MHz, DMSO-*d*_6_) δ 9.57 (s, 1H), 8.64 (s, 1H), 7.57 (s, 1H), 7.41–7.36 (m, 2H), 7.01–6.99 (m, 1H), 1.79 (s, 6H), 1.47 (s, 9H). LC-MS: m/z: 405.1 (M+H)^+^.

### *tert*-Butyl(3-(7-((2-Methoxy-4-(4-methylpiperazin-1-yl)phenyl)amino)-4,4-dimethyl-2-oxo-2*H*-pyrimido[4,5-*d*][1,3]oxazin-1(4*H*)-yl)phenyl)carbamate (19a)

To a mixture of **18a** (1.010 g, 2.5 mmol), 2-methoxy-4-(4-methylpiperazin-1-yl)aniline (0.663 g, 3 mmol) in isopropanol (25 mL) was added trifluoroacetic acid (280 μL, 3.77 mmol). The reaction solution was heated with reflux for 10 h. After cooling to room temperature, the mixture was neutralized with saturated aqueous NaHCO_3_, and extracted with CH_2_Cl_2_ (3 × 40 mL). The organic layer was washed with saturated aqueous NaCl, dried over anhydrous Na_2_SO_4_ and concentrated in vacuo. The residue was purified by silica gel chromatography (ethylacetate/methanol = 25:1, v/v) to give the product **19a** (0.867 g, 59%). ^1^H NMR (400 MHz, CDCl_3_) δ 8.07 (s, 1H), 7.51 (d, *J* = 8.4 Hz, 2H), 7.47–7.43 (m, 2H), 7.01 (d, *J* = 7.2 Hz, 1H), 6.45 (s, 1H), 6.44 (d, *J* = 2.4 Hz, 1H), 6.18–6.16 (m, 1H), 3.82 (s, 3H), 3.16 (t, *J* = 4.4 Hz, 4H), 2.67 (t, *J* = 4.4 Hz, 4H), 2.42 (s, 3H), 1.80 (s, 6H), 1.49 (s, 9H). LC-MS: m/z: 590.4 (M+H)^+^.

### *N*-(3-(7-((2-Methoxy-4-(4-methylpiperazin-1-yl)phenyl)amino)-4,4-dimethyl-2-oxo-2*H*-pyrimido[4,5-*d*][1,3]oxazin-1(4*H*)-yl)phenyl)acrylamide (20a)

To a solution of **19a** (0.500 g, 0.85 mmol) in dichloromethane (10 mL) was added trifluoroacetic acid (2 mL). The mixture was stirred for 5 h at room temperature. The reaction solution was neutralized with saturated aqueous NaHCO_3_, and extracted with dichloromethane (3 × 30 mL). The organic layer was washed with saturated aqueous NaCl, dried over anhydrous Na_2_SO_4_ and concentrated in vacuo to obtain a white solid (0.335 g, 81%), which was used in the next reaction without further purification.

To a solution of the above obtained compound (0.335 g, 0.68 mmol), DIPEA (0.131 g, 1.02 mmol) in CH_2_Cl_2_ (5 mL) was added acryloyl chloride (72 μL, 0.88 mmol) dissolved in CH_2_Cl_2_ (1 mL) at 0 °C. The reaction mixture was stirred overnight at room temperature. The mixture was partitioned between dichloromethane (100 mL) and saturated aqueous NaHCO_3_ (10 mL). The organic layer was washed with saturated aqueous NaCl, dried over anhydrous Na_2_SO_4_ and concentrated in vacuo. The residue was purified by silica gel chromatography (dichloromethane/methanol = 15:1, v/v) to give the product **20a** (0.165 g, 45%). ^1^H NMR (400 MHz, CDCl_3_) δ 8.08 (s, 1H), 7.79 (d, *J* = 8.0 Hz, 1H), 7.74 (s, 1H), 7.47 (t, *J* = 8.0 Hz, 1H), 7.38–7.36 (m, 1H), 7.05 (d, *J* = 7.6 Hz, 1H), 6.41 (d, *J* = 2.0 Hz, 1H), 6.36–6.33 (m, 2H), 6.13 (s, 1H), 5.70 (dd, *J* = 9.2 Hz, *J* = 2.4 Hz, 1H), 3.80 (s, 3H), 3.17 (t, *J* = 4.4 Hz, 4H), 2.80 (t, *J* = 4.4 Hz, 4H), 2.50 (s, 3H), 1.80 (s, 6H). ^13^C NMR (100 MHz, CDCl_3_) δ 155.79, 152.22, 151.27, 139.83, 139.74, 136.17, 131.23, 131.19, 129.68, 127.54, 124.41, 122.18, 122.11, 120.62, 120.54, 120.05, 119.96, 108.34, 100.54, 80.76, 55.61, 54.54, 49.21, 45.09, 28.32. HRMS(ESI) (m/z): (M+H)^+^ calcd for C_29_H_34_N_7_O_4_ 544.2672, found, 544.2698. HPLC purity: 95.77%, retention time = 10.81 min.

The following compounds **20b** and **20c** were prepared by a method similar to that for the synthesis of compound **20a**.

### *N*-(3-(4,4-Diethyl-7-((2-methoxy-4-(4-methylpiperazin-1-yl)phenyl)amino)-2-oxo-2*H*-pyrimido[4,5-*d*][1,3]oxazin-1(4*H*)-yl)phenyl)acrylamide (20b)

^1^H NMR (400 MHz, CDCl_3_) δ 9.42 (s, 1H), 7.96 (s, 1H), 7.89 (s, 1H), 7.81 (d, *J* = 5.6 Hz, 1H), 7.53 (s, 1H), 7.42 (t, *J* = 8.0 Hz, 1H), 7.33–7.31 (m, 1H), 6.99 (d, *J* = 7.6 Hz, 1H), 6.55–6.49 (m, 1H), 6.36–6.32 (m, 2H), 6.12 (d, *J* = 7.6 Hz, 1H), 5.65 (d, *J* = 10.4 Hz, 1H), 3.78 (s, 3H), 3.41 (t, *J* = 4.4 Hz, 4H), 3.21 (t, *J* = 4.4 Hz, 4H), 2.80 (s, 3H), 2.11–1.96 (m, 4H), 1.00 (t, *J* = 7.2 Hz, 6H). ^13^C NMR (100 MHz, CDCl_3_) δ 164.29, 158.75, 156.87, 153.46, 151.27, 148.56, 144.89, 140.04, 136.51, 131.50, 129.71, 127.67, 124.29, 123.12, 120.86, 120.05, 119.46, 108.92, 105.55, 101.12, 87.13, 55.75, 53.61, 47.76, 43.41, 32.88, 7.90. HRMS(ESI) (m/z): (M+H)^+^ calcd for C_31_H_38_N_7_O_4_ 572.2985, found, 572.2981. HPLC purity: 98.86%, retention time = 12.08 min.

### *N*-(3-(7-((2-Methoxy-4-(4-methylpiperazin-1-yl)phenyl)amino)-2-oxo-4,4-dipropyl-2*H*-pyrimido[4,5-*d*][1,3]oxazin-1(4*H*)-yl)phenyl)acrylamide (20c)

^1^H NMR (400 MHz, CDCl_3_) δ 8.06 (s, 1H), 7.96 (s, 1H), 7.77 (d, *J* = 4.8 Hz, 1H), 7.49 (s, 1H), 7.41 (t, *J* = 8.0 Hz, 1H), 6.97 (d, *J* = 8.0 Hz, 1H), 6.40 (d, *J* = 2.0 Hz, 1H), 6.36 (dd, *J* = 16.8 Hz, *J* = 1.2 Hz, 1H), 6.18–6.12 (m, 2H), 5.68 (dd, *J* = 10.4 Hz, *J* = 1.2 Hz, 1H), 3.79 (s, 3H), 3.09 (t, *J* = 4.4 Hz, 4H), 2.58 (t, *J* = 4.8 Hz, 4H), 2.36 (s, 3H), 2.07–2.00 (m, 2H), 1.96–1.89 (m, 2H), 1.54–1.38 (m, 4H), 0.97 (t, *J* = 7.2 Hz, 6H). ^13^C NMR (100 MHz, CDCl_3_) δ 163.56, 158.89, 156.43, 153.34, 151.78, 139.74, 136.05, 131.38, 129.78, 127.26, 124.14, 121.50, 120.46, 120.21, 107.93, 99.92, 86.64, 55.58, 55.05, 49.77, 46.00, 42.86, 16.81, 14.10. HRMS(ESI) (m/z): (M+H)^+^ calcd for C_33_H_42_N_7_O_4_ 600.3298, found, 600.3297. HPLC purity: 99.69%, retention time = 14.41 min.

### *In Vitro* Enzymatic Activity Assay

The enzymatic activity assay was evaluated by using the well-established ELISA-based assay. Kinases domain of EGFR^L858R/T790M^ and EGFR^WT^ were expressed by using the Bac-to-Bac™ baculovirusexpression system (Invitrogen, US), followed by purified in Ni-NTA columns (QIAGEN Inc., US). The detailed experiment was performed according to the published literature^21^.

### *In Vitro* Cellular Antiproliferation Assay

*In vitro* cellular antiproliferation activities of the designed compounds against H1975 and A431 cell lines were examined by using the well-established sulforhodamine B (SRB) colorimetric assay^28^. The detailed experiment was performed according to the published literature^21^. Briefly, H1975 and A431 cells were seeded in 96-well plates, and allowed to adhere overnight followed by treated with gradient concentrations of the test compounds for 72 h. The inhibition rate for cell proliferation was calculated as [1 − (*A*_515_ treated/*A*_515_ control)] × 100%. The IC_50_ values were obtained by using the Logit method and reported as the mean ± SD from three independent determinations.

### Western Blot Analysis

Western blot analysis was performed with standard procedures. Briefly, H1975 and A431 cell lines were seeded in six-well plates, starved in serum-free medium for 24 h, followed by incubation for 2 h in the presence of different concentrations of compounds **20a** and **8** (as positive control). Cells were stimulated by the addition of EGF (50 ng/mL, R&D Systems, US) for 10 min, then washed with PBS and lysed in SDS lysis buffer. Antibodies against p-EGFR (Y1068; #3777 S), EGFR (#4267 S), p-ERK (T202/Y204; #4370 L), ERK (#4695 S), p-AKT (Ser473), AKT and Tubulin were used (Cell Signaling Technologies, Cambridge, MA).

### *In Vivo* Antitumor Efficacy Study

All experimental procedures involving animals were performed in accordance with the ethical guidelines from Shanghai Laboratory Animal Public Service Center and Animal Ethics Committee of the East China University of Science and Technology. The whole experimental protocol was also approved by the Animal Ethics Committee of the East China University of Science and Technology. All 4–6 weeks old nude mice were purchased from Shanghai Slac Laboratory Animal Co. Ltd (The license of experimental animal: SCXK (shanghai) 2012–0002). The mice were maintained under the 12 hour light/dark cycles condition in the specific pathogen free (SPF) cleanroom with fresh air (more than 50%), appropriate temperature (22–26 °C) and humidness (40–60%). All materials and containers were disinfected and sterilized before use. Nude mice, aged 4–6 weeks, were inoculated subcutaneously into the right flank with H1975 NSCLC cells (approximately 2 × 10^6^ cells/mouse). When the tumor size reached a volume of ~200−400 mm^3^, animals were randomly assigned into control and treated groups (n = 3/group). For efficacy studies, mice in control and in treatment groups were treated with vehicle compound **7** and compound **20a** (50 mg/kg/day) for continuous 14 days, respectively. The sizes of tumor volume (V) were measured every two or three days by using vernier caliper, and were calculated with the formula V = (L × W^2^)/2, where L and W stands for length and width, respectively.

## Electronic supplementary material

Supporting Information

## References

[1] Favoni RE, De Cupis A (2000). The role of polypeptide growth factors in human carcinomas: new targets for a novel pharmacological approach. Pharmacol. Rev..

[2] Lemmon MA, Schlessinger J (2010). Cell signaling by receptor tyrosine kinases. Cell.

[3] Mendelsohn J, Baselga J (2003). Status of epidermal growth factor receptor antagonists in the biology and treatment of cancer. J. Clin. Oncol..

[4] Bianco R, Melisi D, Ciardiello F, Tortora G (2006). Key cancer cell signal transduction pathways as therapeutic targets. Eur. J. Cancer.

[5] Levitzki A (2013). Tyrosine kinase inhibitors: views of selectivity, sensitivity, and clinical performance. Annu. Rev. Pharmacol. Toxicol..

[6] Lynch TJ (2004). Activating mutations in the epidermal growth factor receptor underlying responsiveness of non–small-cell lung cancer to gefitinib. N. Engl. J. Med..

[7] Pao W (2004). EGF receptor gene mutations are common in lung cancers from “never smokers” and are associated with sensitivity of tumors to gefitinib and erlotinib. Proc. Natl. Acad. Sci..

[8] Cheng, X. & Chen, H. Tumor heterogeneity and resistance to EGFR-targeted therapy in advanced nonsmall cell lung cancer: challenges and perspectives. *Onco Targets Ther*. **7** (2014). 10.2147/OTT.S66502 PMC4181629 25285017

[9] Tiseo M, Bartolotti M, Gelsomino F, Bordi P (2010). Emerging role of gefitinib in the treatment of non-small-cell lung cancer (NSCLC). Drug Des. Devel. Ther..

[10] Lin, L. & Bivona, T. G. Mechanisms of resistance to epidermal growth factor receptor inhibitors and novel therapeutic strategies to overcome resistance in NSCLC patients. *Chemotherapy research and practice* **2012** (2012). 10.1155/2012/817297 PMC3437267 22970367

[11] Xu Y, Liu H, Chen J, Zhou Q (2010). Acquired resistance of lung adenocarcinoma to EGFR-tyrosine kinase inhibitors gefitinib and erlotinib. Cancer Biol. Ther..

[12] Helena AY (2013). Analysis of tumor specimens at the time of acquired resistance to EGFR-TKI therapy in 155 patients with EGFR-mutant lung cancers. Clin. Cancer Res..

[13] Miller VA (2012). Afatinib versus placebo for patients with advanced, metastatic non-small-cell lung cancer after failure of erlotinib, gefitinib, or both, and one or two lines of chemotherapy (LUX-Lung 1): a phase 2b/3 randomised trial. The lancet oncology.

[14] Giaccone G, Wang Y (2011). Strategies for overcoming resistance to EGFR family tyrosine kinase inhibitors. Cancer Treat. Rev..

[15] Reckamp KL (2014). A phase 2 trial of dacomitinib (PF‐00299804), an oral, irreversible pan‐HER (human epidermal growth factor receptor) inhibitor, in patients with advanced non–small cell lung cancer after failure of prior chemotherapy and erlotinib. Cancer.

[16] Zhou W (2009). Novel mutant-selective EGFR kinase inhibitors against EGFR T790M. Nature.

[17] Walter AO (2013). Discovery of a mutant-selective covalent inhibitor of EGFR that overcomes T790M-mediated resistance in NSCLC. Cancer Discov.

[18] Cross DA (2014). AZD9291, an irreversible EGFR TKI, overcomes T790M-mediated resistance to EGFR inhibitors in lung cancer. Cancer Discov.

[19] Steuer CE, Khuri FR, Ramalingam SS (2015). The next generation of epidermal growth factor receptor tyrosine kinase inhibitors in the treatment of lung cancer. Cancer.

[20] Greig SL (2016). Osimertinib: First Global Approval. Drugs.

[21] Hao Y (2016). Discovery and Structural Optimization of N5-Substituted 6, 7-dioxo-6, 7-dihydropteridines as Potent and Selective Epidermal Growth Factor Receptor (EGFR) Inhibitors Against L858R/T790M Resistance Mutation. J. Med. Chem..

[22] Zhou W (2013). Discovery of Pteridin-7 (8 H)-one-based irreversible inhibitors targeting the epidermal growth factor receptor (EGFR) Kinase T790M/L858R mutant. J. Med. Chem..

[23] Glide, version 5.5 Schrödinger, LLC, New York, USA. www.schrodinger.com (2009).

[24] Maestro, version 9.0 Schrödinger, LLC, New York, USA. www.schrodinger.com (2009).

[25] Gong J (2013). ChemMapper: a versatile web server for exploring pharmacology and chemical structure association based on molecular 3D similarity method. Bioinformatics.

[26] Liu X, Jiang H, Li H (2011). SHAFTS: a hybrid approach for 3D molecular similarity calculation. 1. Method and assessment of virtual screening. J. Chem. Inf. Model..

[27] Laskowski RA, Swindells MB (2011). LigPlot+: Multiple Ligand–Protein Interaction Diagrams for Drug Discovery. J. Chem. Inf. Model..

[28] Li M (2016). DW10075, a novel selective and small-molecule inhibitor of VEGFR, exhibits antitumor activities both *in vitro* and *in vivo*. Acta Pharmacologica Sinica.

