# Supplementary material for: Structure-Guided Design of C4-alkyl-1,4-dihydro-2H-pyrimido[4,5-d][1,3]oxazin-2-ones as Potent and Mutant-Selective Epidermal Growth Factor Receptor (EGFR) L858R/T790M Inhibitors
Source: Sci Rep. 2017 Jun 19;7:3830. doi: 10.1038/s41598-017-04184-9 (PMC5476563; doi:10.1038/s41598-017-04184-9)
Supplement: Supplementary file 1 — Supporting Information [file 41598_2017_4184_MOESM1_ESM.pdf]

Supporting Information

Structure-Guided Design of C4-alkyl-1,4-dihydro-2H-pyrimido[4,5-d][1,3]oxazin-2-ones as Potent and Mutant-Selective Epidermal Growth Factor Receptor (EGFR) L858R/T790M Inhibitors

Yongjia Hao[‡,1] Jiankun Lyu[‡,1] Rong Qu[‡,2,3] Deheng Sun,[1] Zhenjiang Zhao[1], Zhuo Chen[1], Jian Ding[2], Hua Xie[2,]*, Yufang Xu[1, *] and Honglin Li[1, *]

[1]*State Key Laboratory of Bioreactor Engineering, Shanghai Key Laboratory of New Drug Design, School of Pharmacy, East China University of Science & Technology, Shanghai 200237, China.* [2]*Division of Anti-tumor Pharmacology, State Key Laboratory of Drug Research, Shanghai Institute of Materia Medica, Chinese Academy of Sciences, Shanghai, 201203, China.* [3]*University of Chinese Academy of Sciences, Beijing 100049, China.*

‡ Authors contributed equally to this work

* To whom correspondence should be addressed. E-mail: hxie@jding.dhs.org, yfxu@ecust.edu.cn, hlli@ecust.edu.cn

Content

Supplementary Figure S1. Source blot data for Figure 5..S3
Supplementary Figure S2. ^{1}H NMR of compound 20a. S4
Supplementary Figure S3. ^{13}C NMR of compound 20a. S4
Supplementary Figure S4. HPLC of compound 20a. S5

Supplementary Figure S1. Source blot data for Figure 5.

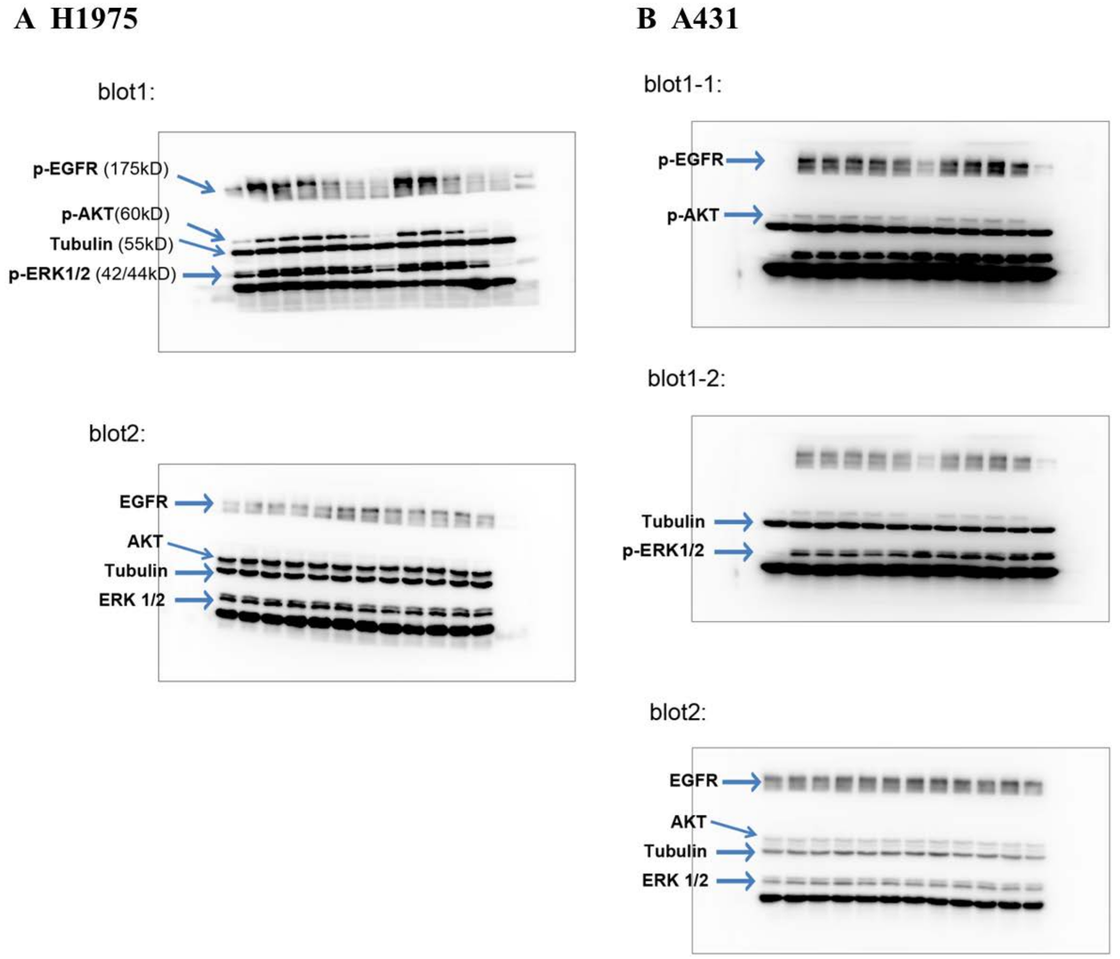

Supplementary Figure S2. ^{1}H NMR of compound **20a**.

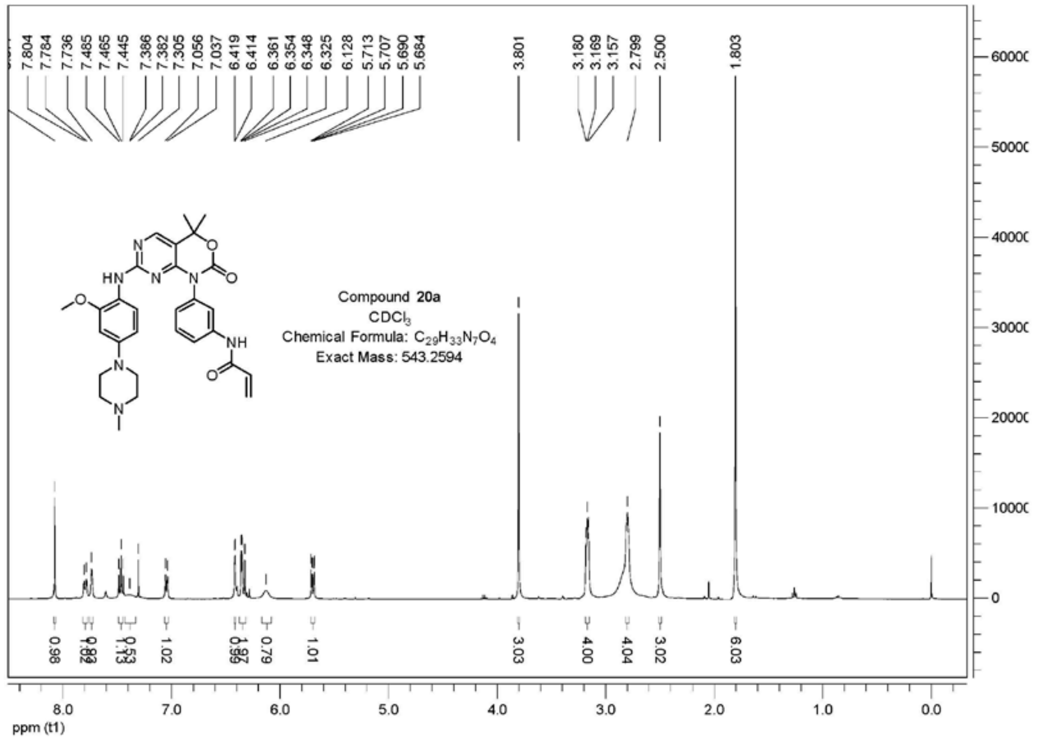

Supplementary Figure S3. ^{13}C NMR of compound **20a**.

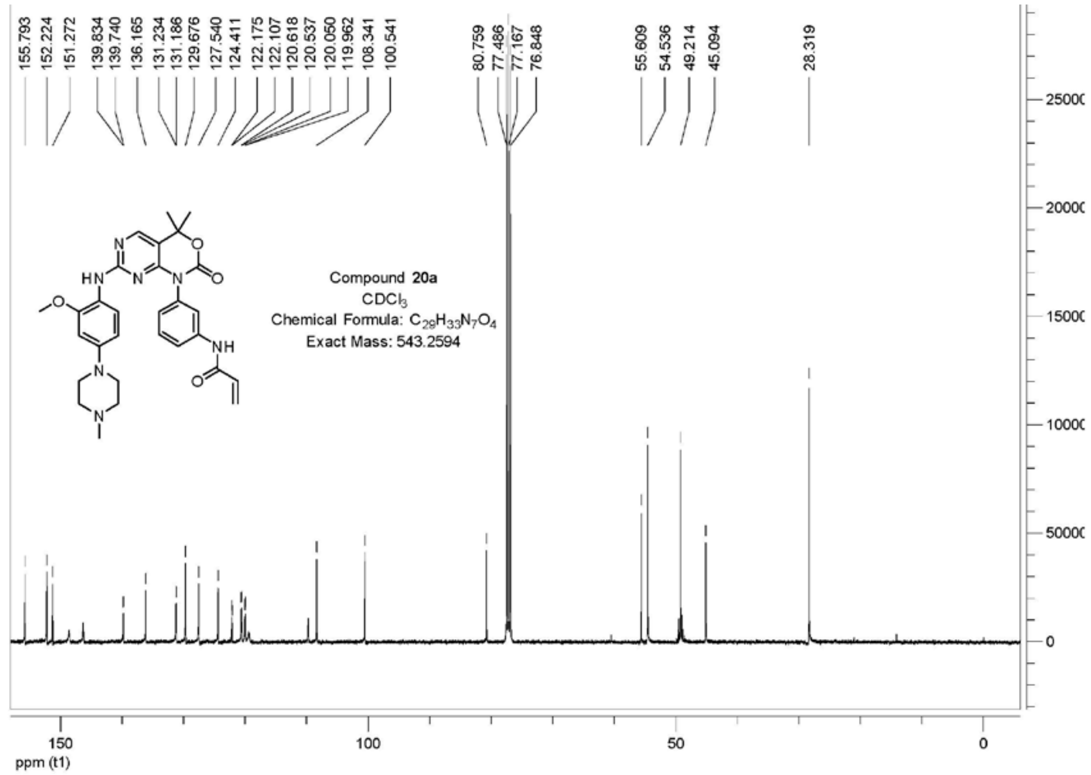

Supplementary Figure S4. HPLC of compound **20a**.

数据文件: E:\HPLCDATA\20151025\FUMEIQ10 2015-10-25 14-15-10\017-2001.D
样品名称: 6号

==

操作者 : MT 序列行 : 20
仪器 : 仪器 1 位置 : 样品瓶 17
进样日期 : 2015/10/25 21:56:38 进样次数 : 1
进样量 : 10.000 µl
采集方法 : D:\CHEM32\1\DATA\FUMEIQ10 2015-10-25 14-15-10\FUMEIQ10-10-2.M
最后修改 : 2015/10/25 18:48:31 : MT
分析方法 : D:\液相HPLC\2\METHODS\1-8.M
最后修改 : 2015/10/31 16:52:07

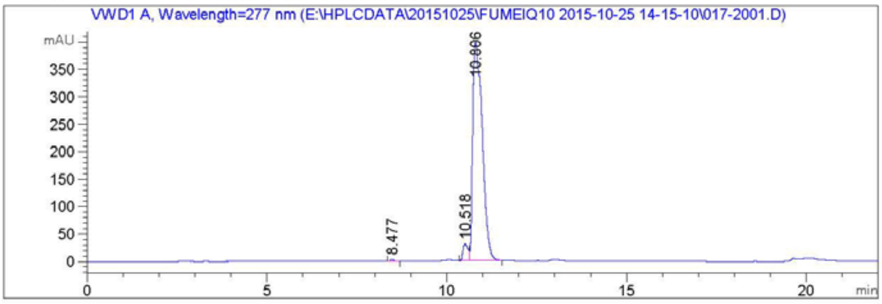

==

面积百分比报告

==

排序 : 信号
乘积因子: : 1.0000
稀释因子: : 1.0000
内标使用乘积因子和稀释因子

信号 1: VWD1 A, Wavelength=277 nm

峰 #	保留时间 [min]	类型	峰宽 [min]	峰面积 [mAU*s]	峰高 [mAU]	峰面积 %
1	8.477	BBA	0.1126	24.75025	3.58316	0.3226
2	10.518	BV	0.1549	299.58649	30.42372	3.9044
3	10.806	VV	0.2556	7348.72412	397.64838	95.7730
总量 :				7673.06086	431.65525	

==

*** 报告结束 ***

仪器 2 2015/10/31 16:52:08 页 1/1
